# Passing the test of motherhood? Self‐esteem development and birth experience in the transition to motherhood: A longitudinal mixed methods study in Finland

**DOI:** 10.1111/jan.15468

**Published:** 2022-10-17

**Authors:** Mirjam Raudasoja, Katri Vehviläinen‐Julkunen, Asko Tolvanen

**Affiliations:** ^1^ Department of Psychology University of Jyväskylä Jyväskylä Finland; ^2^ Department of Nursing Sciences University of Eastern Finland and Kuopio University Hospital Kuopio Finland; ^3^ Methodology Center for Human Sciences University of Jyväskylä Jyväskylä Finland

**Keywords:** childbirth experience, longitudinal study, midwife, mixed method, nursing, self‐esteem

## Abstract

**Aims:**

To investigate women's childbirth experiences and their relation to self‐esteem development in the postpartum year.

**Design:**

A mixed methods study.

**Methods:**

Women (*N* = 125) completed survey questionnaires regarding their self‐esteem and childbirth experiences at three time points in 2020–2021: third trimester of pregnancy (T1), 4–8 weeks postpartum (T2) and 1 year postpartum (T3). The survey results were analysed using qualitative thematic and quantitative path analyses with latent change factors. The open‐ended answers of the women who demonstrated a change in self‐esteem between T2 and T3 were then compared. The STROBE checklist was used as the reporting guideline.

**Results:**

The quantitatively measured childbirth experiences predicted statistically significantly and positively the changes in self‐esteem in the following year. The women described their childbirth stories through three main themes: childbirth as a lived experience, childbirth as a relational event and childbirth as a medical event. On the basis of the thematic analysis, we propose that the relationship between childbirth experience and self‐esteem development might only hold for women with extremely positive or negative childbirth experiences. There were mixed results for those women who had mixed experiences, indicating that other factors probably contributed to the changes in self‐esteem.

**Conclusion:**

Childbirth is a pivotal event that may have lasting effects on the mother's self‐esteem after childbirth. Especially women with traumatic experiences deserve attention because they are at risk of the most negative consequences.

**Impact:**

Perinatal services and policy makers must recognize the importance of childbirth experiences in women's well‐being and improve their practices. Different cultural models of childbirth should be recognized and supported to facilitate good experiences and prevent traumatic ones.

**Patient or Public Contribution:**

Service users recruited in Finnish Child Health Centers responded to surveys that were used as data for this study.

**Impact statement**

- Patients will benefit from the results of this study if they are implemented in care systems. For example, mothers will receive better support after traumatic childbirth experiences.
- The results will be useful in clinical practice. They help to identify patients in need of support, because the risks of negative experiences of childbirth are better known.
- The results can be used in training of midwives, doctors, and nurses in perinatal care.
- The results can be used in communicating the importance of the perinatal period to the wider public, which may result in cultural change towards more supportive environment for mothers.

## 1 INTRODUCTION

Childbirth experiences are multifaceted, an integral part of which are formed by feelings of capacity (Dencker et al., 2020). Women describe and evaluate their birthing performances against their own expectations (Preis, Lobel, et al., 2019) and cultural images of childbirth (Davis‐Floyd, 2001; Hall, 2016). These evaluations sometimes produce feelings of accomplishment or failure, which may contribute to mothers' self‐esteem later, supporting or hindering it. Feelings of inadequacy or failure related to childbirth (Schneider, 2013, 2018) or perceived discrepancy between expectations and reality (Preis, Lobel, et al., 2019) may negatively affect women's self‐esteem in early motherhood. On the other hand, feelings of achievement and empowerment (Olza et al., 2018; Simkin, 2006) may promote increased self‐esteem. The quality of the birthing experience has consequences to mothers' perceptions of themselves and their babies (Reisz et al., 2015). However, a gap exists in the current literature on the effect of childbirth experience on self‐esteem. Most studies have concentrated on context‐specific maternal self‐esteem (Laney et al., 2014) or self‐efficacy (Reisz et al., 2015) in the transition to motherhood. Furthermore, studies proposing that childbirth experience might affect maternal self‐esteem later rarely combined quantitative and qualitative data. For this reason, the kinds of childbirth experiences that might affect women's self‐esteem later are not exactly known. The present study examined the effects of childbirth experience on mothers' self‐esteem, combining quantitative and qualitative methods to acquire knowledge on different developmental pathways.

### 1.1 Background

The transition to motherhood involves bodily changes, psychological adaptation, and a changing social environment and can have both positive and negative implications for the mother (Taubman‐Ben‐Ari et al., 2009). A time for relational change, this transition invokes changes in women's self‐concept (Laney et al., 2014). Childbirth is a possibility for psychological growth (Taubman‐Ben‐Ari et al., 2009) but sometimes leads to traumatization and consolidates negative perceptions of oneself (Byrne et al., 2017). Childbirth experiences refer to subjective experiences of labour and childbirth. Larkin et al. (2009) defined childbirth experience as individual life events with mutually related physiological and psychological processes influenced by contexts such as social, environmental and organizational factors. The central components of childbirth experiences include feelings of capacity, perceived safety and security, professional support and participation (Dencker et al., 2020).

One important aspect affecting how women understand and narrate their childbirth experiences is the surrounding culture. Western cultures are often proposed to alternate between two extremes or narratives, natural and medical understandings of childbirth (Preis, Lobel, et al., 2019). Davis‐Floyd (2001) proposed that American culture encompasses three paradigms of childbirth: The technocratic model is built on the separation of the mind and the body and depicts the body as a machine. The body is understood as prone to fail and in need of interventions, and healing is believed to occur outside in. The humanistic model, in turn, stresses a mind–body connection and values the connection and caring between labouring woman and healthcare practitioners. Healing is thought to occur both from the inside out and outside in, and practitioners must know how to listen to women. The holistic model stresses the oneness of body–mind‐spirit and understands the body as an energy system interlinked with other energy systems. Thus, the purpose of healing is to care for the whole person in their whole life context, and healing is thought to occur inside out (Davis‐Floyd, 2001). How women relate to cultural understandings of childbirth may affect how they assess their own labour performances. For example, when a mother has a strong conviction that natural childbirth is best for the child, birthing by caesarean section may cause feelings of failure or inadequacy.

Psychoanalytically oriented researchers have tried to explain how childbirth relates to female psychosexual development. Hall (2016) summarized the results of her analysis of interviews with 30 first‐time mothers before and after their deliveries and proposed three central themes encompassing women's expectations and experiences of childbirth: fear of bodily damage, pride and awe about producing a baby and true womanhood. These themes are often manifested as performance anxiety regarding childbirth choices and mothering (Hall, 2016). Women often experience pressure towards ‘soft’ and ‘natural’ childbirth choices, which may be perceived as indicators of true womanhood (Hall, 2016). However, in modern obstetric settings, childbirths are often more medicalized than expected (Preis, Eisner, et al., 2019). Unmet expectations expose women to feelings of failure that they most often blame themselves for (Schneider, 2010). In turn, at the core of a satisfying childbirth experience is the belief in one's ability to give birth (Olza et al., 2018). Confirming or disconfirming this belief in childbirth may have positive or negative consequences to self‐esteem.

Self‐esteem refers to a person's subjective evaluation of self‐worth (Orth & Robins, 2014). Developed in childhood, self‐esteem is thought to form a basis for psychological well‐being and success in life (Orth & Robins, 2014). In the transition to motherhood, studies have attempted to identify a normative pattern of change in self‐esteem. In a Norwegian study (van Scheppingen et al., 2018) in women who had had their first, second, third or fourth child, it was found that in all subgroups, self‐esteem tended to decrease during pregnancy, increase until the child was 6 months old, and gradually decreased over the following years. In other studies (e.g. Bleidorn et al., 2016), a sharp decrease in self‐esteem has been reported after the birth of the baby, followed by a gradual decrease in the following years. However, these studies have not predicted changes in self‐esteem on the basis of childbirth‐related variables.

Studies that explored the relationship between childbirth experience and self‐esteem have often concentrated on context‐specific maternal self‐esteem (e.g. Laney et al., 2014; Reisz et al., 2015). For example, Reisz et al. (2015) found that subjective childbirth experience predicted context‐specific maternal self‐esteem in mothers who had given birth during the previous year. However, whether general self‐esteem can also be affected by childbirth experience is currently unknown. Qualitative studies that examined the effect of childbirth experience on the mother suggest that good experiences promote a sense of accomplishment (Simkin, 2006) and empowerment (Olza et al., 2018), whereas traumatic experiences challenge and alter women's self‐perceptions (Byrne et al., 2017). Altered self‐perceptions may initiate changes in self‐esteem resulting from a childbirth experience. According to Schneider (2010), an important central theme in childbirth experiences is that ‘birth says something about me’ (p. 104). Women in that study perceived childbirth as transformative and indicative of their inner qualities that they were not always aware of prior to childbirth (Schneider, 2010). Thus, studies suggest that the quality of the childbirth experience may affect women's self‐esteem. However, which types of interpretations must be made about childbirth to induce changes in self‐esteem remain unknown. Integrating quantitative and qualitative data into a mixed methods analysis will improve the existing knowledge about the processes of self‐esteem development. The Finnish maternity system is especially suitable for this kind of study because public, low‐cost maternity services are available to everyone and socioeconomic status does not affect availability of services. This knowledge will help to improve maternity services in Finland and internationally to recognize the impact of self‐esteem for women's well‐being in the perinatal period.

## 2 THE STUDY

### 2.1 Aims

The aim of the present study was to investigate variations in self‐esteem development and their relations to childbirth experience. The research questions were as follows:

1. Does subjective childbirth experience predict changes in self‐esteem during the first year after giving birth?
2. How do women describe their childbirth experiences?
3. How do women's descriptions of their childbirth experiences differ between different groups on the basis of changes in self‐esteem during the first year after giving birth?

### 2.2 Design

**FIGURE 1:**
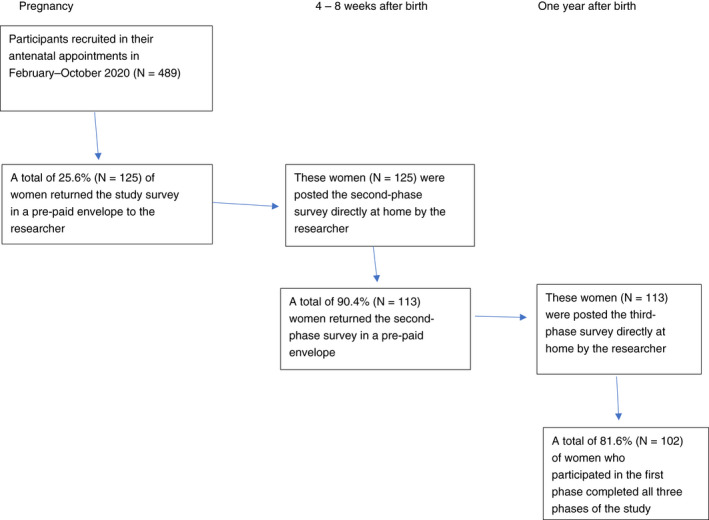
Study design.

In this study, we used a mixed methods approach and longitudinal design. We conform to the STrengthening the Reporting of OBservational studies in Epidemiology (STROBE) standards (see Supplementary File 1). Quantitative and qualitative data were collected through surveys conducted from 2020 to 2021. The study included three measurement points: the third trimester of pregnancy (weeks 30+), 4–8 weeks postpartum and 1 year postpartum. Qualitative data about childbirth experiences were collected at time point 2, whereas quantitative data were collected at all time points. In the first phase of the study, the participation rate was 25.6%. A total of 90.4% (*N* = 113) of women who participated in the first phase also returned the questionnaire in the second phase. A total of 81.6% (*N* = 102) of the women who participated in the first phase completed all three phases of the study. The study design is presented in Figure 1.

This was a mixed methods study using a triangulation design with a three‐phase approach (Creswell & Plano Clark, 2011, p. 62). In the first phase, the quantitative component provided knowledge of whether childbirth experiences predicted changes in self‐esteem during the following year. The results of the first phase informed the second phase (Creswell & Plano Clark, 2011), which was a qualitative investigation needed to provide understanding about how women describe their childbirth experiences. The analysis methods were performed separately, using the quantitative analysis to first answer the subquestion about the predictive value of childbirth experience in self‐esteem development and then using the qualitative analysis to address how the participants described their experiences. Through the integration of the results, we identified the types of experiences that predicted statistically significant changes in the participants' self‐esteem.

### 2.3 Assessment measures

#### 2.3.1 Self‐esteem

Self‐esteem was assessed using the Rosenberg Self‐Esteem Scale (Rosenberg, 1989), which measures the general level of self‐esteem in all three time points. The scale included 10 items such as ‘.’ The statements were answered on a 5‐point Likert scale (1 = strongly disagree; 5 = strongly agree), and items that indicated low self‐esteem were reverse‐scored. The maximum total score on the scale was 50, and higher scores indicated better self‐esteem. The value of Cronbach's alpha for the scale was excellent: 0.91 at T1, 0.90 at T2 and 0.90 at T3.

#### 2.3.2 Childbirth experience

The quality of childbirth experience was measured using the Delivery Satisfaction Scale (DSS) between 4 and 8 weeks postpartum. The DSS is an eight‐item scale developed and validated in Finland (Saisto et al., 2001). The scale was answered on a 5‐point Likert scale, and all but two items (questions 4 and 8) were reverse‐scored. It consisted of items such as ‘Was childbirth a positive experience for you’? (1 = very; 5 = not at all). The maximum total score was 40, and a higher score indicated a more positive experience. The value of Cronbach's alpha for the scale was good, 0.78.

The quality of childbirth experience was also measured with an open‐ended question at the beginning of the questionnaire: ‘How was your experience of birth? Describe freely’. Below the question was a blank space of nine lines to write on.

### 2.4 Participants

The study participants were recruited in four medium‐sized cities in Central Finland using convenience sampling. In the area, a total of 2754 women gave birth in the year 2020. Women were eligible to participate in the study if they were at least 30 weeks into gestation and could complete the survey in Finnish. Prior to the data collection, we expected to have at least 100 participants. A total of 489 women received the study survey questionnaire, and 125 women were enrolled in the study.

### 2.5 Data collection

Participants were recruited via public health nurses in family health centres in their antenatal appointments between February and October 2020. They were provided with oral and written information about the study, a voluntary participation form, and the study survey questionnaire. They answered the survey questionnaire at home and returned it to the researcher in a prepaid envelope. In the second phase, the women who had participated in the first phase were approached directly by mail and asked to complete a second questionnaire and to return it to the researcher in a prepaid envelope. The third‐phase survey questionnaire was sent to the women who participated in both the first and second phases, and they were asked to complete the questionnaire and send it to the researcher in a prepaid envelope. Participants were sent two text messages at 1‐month intervals reminding them to participate if they had not completed the surveys on time. Reasons for not participating or dropping out were not asked for.

### 2.6 Ethical considerations

Before data collection, ethical approval for the study was obtained from the ethics committee of the University of Jyväskylä (August 2019) and the study conforms to the Declaration of Helsinki standards. All participants received oral and written information about the study. Participation was voluntary and could be stopped at any time without consequences for the participant. As the study survey included questions about potentially sensitive experiences, all participants were provided with a chance to contact the responsible researcher to discuss any thoughts or feelings that participation might invoke. All data were anonymized and stored in a safe repository during data collection. All data samples are presented under pseudonyms.

### 2.7 Data analysis

The first step of the analysis was quantitative, consisting of a path analysis with latent change factors (Voelkle & Oud, 2015). By modelling latent change factors both the mean changes, as well as individual variations, across these means are estimated. Three latent factors, namely F1, F2 and F3, for each self‐esteem variables at time T1, T2 and T3 were specified, and factor loadings were set to one. The model was identified when residual variances were set as equal at the T1, T2 and T3 measurements. In this way, we could isolate the measurement error. Paths from F1 to F2 and from F2 to F3 were set at one, and the latent change factors CHF2 and CHF3 were set to capture all residual variances from F2 to F3. Therefore, the residual variances of F2 and F3 were set to zero. The paths from F1 to CHF2 and from F2 to CHF3 were freely estimated. Childbirth experience was regressed on self‐esteem at T1, and change in self‐esteem at T3 was regressed on childbirth experience. The saturated model was estimated using the full information maximum likelihood method with the Mplus 8.6 statistical program (Muthén & Muthén, 1998–2017). Standard errors were estimated with the ‘maximum likelihood with robust standard errors’ estimator, whose estimates are robust against non‐normal distributions. Missing values (10% at T2 and 19% at T3) were supposed to be random, actually, the Little's MCAR test $\chi^{2}\left(7\right)=13.22,\;p=.067$ showed that the assumption of ‘missing completely at random (MCAR)’ cannot be rejected.

The second step of the analysis was to investigate responses to the open‐ended question at T2, considering childbirth experience. All responses (*N* = 113) were analysed by the first author using a thematic analysis (Braun & Clarke, 2006) to determine how childbirth experiences are constituted in women's open‐ended responses. At first, the data were read through several times, and initial thoughts were written down. Then, the first half of the data were analysed for repetitive meanings, words, and sentences that were highlighted to obtain interesting and significant details from the data. Next, initial themes were formulated using the second half of the data, and these themes were then grouped under higher‐order themes. The themes identified were tested against the first half of the data: a few lower‐order themes were identified, and the higher‐order themes remained the same. Finally, the responses were read as whole stories to determine what childbirth experiences mean to the participants.

After the statistical and thematic analyses, we selected individuals who showed a statistically significant change (*p* < .05) in their self‐esteem mean score between T2 and T3 (*n* = 14). The individuals were divided into four groups: (1) those with a positive childbirth experience with increasing self‐esteem; (2) those with a negative childbirth experience with an increasing self‐esteem; (3) those with a negative childbirth experience with a decreasing self‐esteem and (4) those with a positive childbirth experience with a decreasing self‐esteem. This grouping was done based on the qualitative responses concerning the childbirth experience (the general tone and adjectives used to describe the birth experience). After that, the correspondence with DSS scores was checked. It seemed that all positive descriptions were for individuals whose DSS mean score was 4 or more; all negative descriptions were for individuals whose DSS score was less than 4. The responses of the four different groups to the open‐ended question on childbirth experience were compared with each other.

### 2.8 Validity and reliability

The statistical model was saturated, and all the instruments showed very good reliability. The statistical power to detect small effects at 0.05 level (0.27) with the planned sample size n = 100 was 0.80. This sample size is considered sufficient to find significant effects. In qualitative analysis, a second coder (KVJ) coded every tenth answer to ensure the validity of the thematic structure. Analytic decisions and disagreements were discussed between the researchers until consensus was reached. The themes were further refined following the discussions. For example, the hierarchy of some of the themes changed after these reflections. An audit trail was kept during the analytical phase, including analytical decisions, the evolving thematic structure, discussions between the researchers, and the first analysts' thoughts and feelings about the subject. The findings of the statistical analyses and their relations to the qualitative data were actively discussed between the researchers.

## 3 FINDINGS

### 3.1 Sample

The study participants were 20 to 46 years old (mean [*SD*] age: 31 [4.49] years). Seventy‐three participants (58.4%) were primiparous, and 52 (41.6%) were parous. The mean age was representative of all Finnish childbearing women, but the proportion of primiparous women was slightly greater. On average, the participants were highly educated. Details of the sample are presented in Table 1. Participants reported, on average, a mean DSS score of 4.02 (*SD* 0.62), indicating a positive experience of birth at T2 (see Table 1). The mean sum score of RSES was also high, indicating a high self‐esteem, on average: 40.39 (*SD* 7.18) at T1, 40.49 (*SD* 7.25) at T2 and 41.27 (*SD* 6.96) at T3 respectively. However, big standard deviations indicated a large individual variation in both variables.

**TABLE 1:** Background characteristics of the sample (*n* = 125)

	*M* (*SD*)	*n* (%)
Age	31.1 (4.46)	
Number of children	0.62 (1.02)	
Primiparous		73 (58.4)
Parous		52 (41.6)
Level of education		
University or college degree		90 (72.0)
Technical college degree		33 (26.4)
No formal education after compulsory schooling		1 (0.8)
Missing data		1 (0.8)
Family form		
Nuclear family		115 (92.1)
Blended family		8 (6.8)
Other		2 (1.8)
Perceived financial situation		
Better than average		27 (21.6)
Average		86 (68.8)
Poorer than average		10 (8.0)
Poor		1 (0.8)
Missing data		1 (0.8)
Self‐esteem (RSES sum score)		
T1	40.39 (7.18)	
T2	40.49 (7.25)	
T3	41.27 (6.96)	
Childbirth experience (DSS mean score)		
T2	4.02 (0.62)	
T3	3.98 (0.64)	

*Note*: Marital status and religious orientation of the participants were not asked about, nor were gender identities, sexual orientation or (dis)abilities.

### 3.2 Childbirth experiences and self‐esteem

**FIGURE 2:**
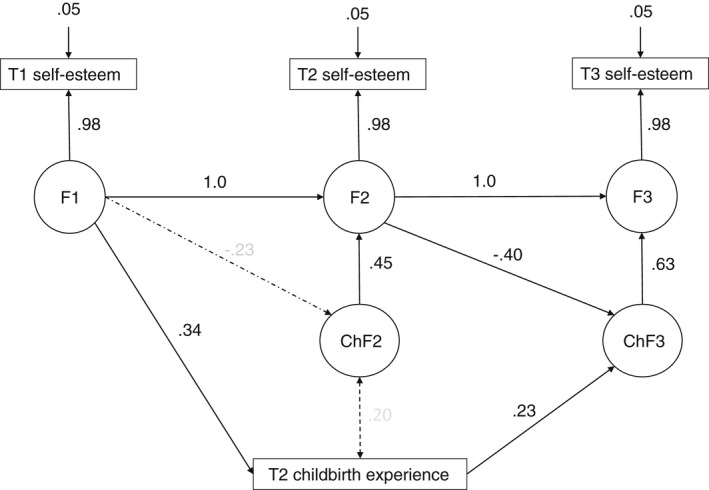
Relationship between self‐esteem, childbirth experience and change in self‐esteem.

The first research question was whether childbirth experience predicts changes in self‐esteem during the first year after childbirth. Self‐esteem at T1 was positively associated with childbirth experience at T2; that is, the higher the self‐esteem at T1, the more positive the childbirth experience at T2. Childbirth experience at T2 was positively associated with change in self‐esteem between T2 and T3, which means that the more positive the childbirth experience at T2, the higher the increase in self‐esteem from T2 to T3 (see Figure 2). Maternal age, parity, income level and education level did not correlate statistically significantly with childbirth experience and self‐esteem and were therefore left out of the model. Numbers for missing values were: self‐esteem T1 *n* = 0, T2 *n* = 12, T3 *n* = 23 and childbirth experience T2 *n* = 12. At the 0.80 statistical power, we are able to detect 0.25, 0.26 and 0.27 standardized regression coefficient when the sample size is 125, 113 and 102 respectively. If the effects are smaller, it is difficult to find them with these sample sizes.

### 3.3 Childbirth experiences

The second research question was how women describe their childbirth experiences. All women who returned the survey at T2 (*n* = 113) responded to the open‐ended question. As a result of the thematic analysis of the open‐ended responses on childbirth experience, nine themes were identified. These themes were grouped under three overarching themes that capture the meaning of childbirth as constructed by the participant: (1) childbirth as a lived experience; (2) childbirth as a relational event and (3) childbirth as a medical event. Often, more than one overarching theme was present in one answer. A thematic structure is presented in Table 2.

**TABLE 2:** Presentation of the thematic analysis

Overarching theme	Lower‐level themes and their contents	Data examples
Childbirth as a lived experience	Cognitive and emotional appraisal: describing the experience, comparing with a previous childbirth or with expectations, feelings considering birth, appraising the decisions afterwards, evaluating the experience on a scale, possible future births Reactions and actions during birth: control/ diminished control, decision making, enduring, childbirth sensations, emotions, moments of disorientation Situating childbirth in the life story: Before the childbirth: feelings (anxiety/fear), expectations, hopes, preparing for birth, expectations for partner, other life events; birth experience as compared with previous births; changing perceptions over time; after the birth	*It wasn't like I expected. […] I expected a calmer and more controllable experience in terms of pain, I did not experience it a very natural event because of medical induction and pain relief. At first, I was upset of my experience, now [I'm] mostly proud that I managed a difficult birth*. *During labor, I realized that I was bad at enduring pain. For that reason, opening phase was difficult, but I do not remember it in a bad way anymore. The pushing stage was OK for me because I felt that then I could best affect it [the labor] myself*. *Afterwards I am disappointed, on the other hand, because I did not have opportunity to experience a ‘normal’ birth, for I waited it for 9 months if not longer*. *I could birth again whenever*.
Childbirth as a relational event	4 Relationships with family and known people: relationship with baby (meeting the baby, describing the baby); relationship with partner (support from the partner, trusting the partner); relationship with another familiar person (someone familiar is present at birth, doula could be mentioned); becoming a family (cooperation with partner, first moments as a family) 5 Relationships with professionals: experiences of care or neglect, trust, ‘chemistry’ between mother and midwife, interaction, information	*I received a lot of support from my husband, and I experienced that our relationship was strengthened. Maybe a birth that went well also helped me to fall in love with the baby straight away. The first moments as a family were very meaningful*. *I had a strong sense of security, also a feeling that I and the father of the child (who was present at birth, in the birth room), gave birth to the child together*. *I had the water birth I hoped for, with me my partner, a doula (who is also a good friend) and a midwife. I trusted myself and everybody else seemed to trust my body and it indeed worked like automatically*.
Childbirth as a medical event	6 Obstetrical story: the duration of the childbirth stages and childbirth events, parity, gestational age, complications, interventions (induction, augmentation, episiotomy, ventose), pain relief, size of the baby, mode of birth, ‘natural birth’ 7 Condition of the baby (heartbeat during labour, condition after childbirth, Apgar score), 8 Condition of the mother (after the childbirth) 9 The care system: transitions (home to hospital, reception to delivery suite, delivery suite to maternal ward, hospital to home), aspects and quality of care (positive/negative evaluation), controlling power of the hospital system (corona restrictions, transitions ‘allowed’, obstetric violence)	*My labor lasted about 11 h, beginning from arrival at hospital at 1000 h noon for a scheduled induction and ending at the birth of my daughter at 2100 h in the evening. I did not have contractions before they ruptured the membranes. The labor was fairly quick even though contractions continued quite a while, so I got an epidural [anaesthesia] for pain relief. I suffered from slight dryness because nothing kept inside, and I was not allowed to drink for the risk of the bladder impeding the labor. The child was 3.9 kg, [and it] came head first with ventose [extraction]. […] I would imagine that the labor went in a usual way. The heartbeat of the child became weaker a couple of times, and it took a long time before we got a midwife to come. We started to be afraid for the well‐being of the child. The child did not descend [in the pelvis] and they did not intervene because of the hurry of midwives before 2 h. Finally, we ended up in fierce ventose extraction*. *The midwives did not tell that they were going to do an episiotomy, I realized myself when they spoke together about anaesthesia*.

#### 3.3.1 Childbirth as a lived experience

Childbirth as a lived experience was described through three lower‐level themes: (1) cognitive and emotional appraisal; (2) reactions and actions during childbirth and (3) situating birth in the life story. Cognitive and emotional appraisal consisted of several subthemes: describing the experience with various adjectives (e.g. great, good, hard, fearful and horrible), describing how one feels about childbirth afterward, comparing the experience with a previous childbirth or with expectations, appraising the decisions made during childbirth, and evaluating the experience on a scale (e.g. ‘I rated the experience 10/10’). Emma (28 years old, first child) expressed her overall evaluation as follows:

The birth was long and painful. […] I received all the pain relief, but in the end, nothing helped anymore. It hurt terribly. […] Now that I think of the birth, I wonder if it was a nightmare. It did leave me with fear of childbirth!

Emma's description indicated an overwhelming impression of pain. So difficult did Emma experience labour that she even wondered ‘if it was a nightmare’. The impression in her story was that labour was something that happened to her, leaving her with intolerable pain and fear of childbirth to carry to the future.

The second theme in this category was one's reactions and actions during childbirth. The participants described them through several subthemes: feelings of control or diminished control, decision making, ‘enduring’ it, childbirth sensations, emotions and moments of disorientation. Raisa (39 years old, second child) wrote:

This time, the birth was very empowering and great experience! I gave birth without pain relief, and for that purpose, I had learned to relax well. When I was bathing in the sauna in our sauna building on the yard and when I was dancing on the terrace of the sauna to good music, I felt downright euphoric! I could not have imagined that anybody could feel that good while having contractions every second minute or a little bit more than one minute. […] In addition, right after the birth, I was totally in control: I told my husband to help me out of my bra so that I could take the baby to the breast. I instructed the midwives to let the cord pulse as long as it would, and I refused the oxytocin drip, because there was no immediate need for that (I asked that).

This very positive childbirth experience included many descriptions of Raisa's own reactions and actions during labour. Feelings of control were apparent: having learnt how to relax, bathing in the sauna, dancing, concentrating, being in control, and instructing husband and midwives. Childbirth sensations were mainly depicted as positive, and Raisa even felt ‘euphoric’, which she found somewhat interesting herself. Raisa also described herself as in charge of decision making, which strengthened the impression of being in control.

Some participants described their childbirths as part of a life story, describing their experiences over time. They described several aspects from before the childbirth: feelings, anxieties and fears, expectations, hopes, preparation for childbirth, expectations for partner and other life events. They also considered their experiences of previous childbirths and anticipated possible future childbirths (stating that they could give birth again, doubting it or expressing that they never want to give birth again). Jenni (27 years old, first child) wrote:

I feel that I exceeded myself. The experience is now an extremely dear memory for me, and it gives me strength.

This positive experience captured how childbirth experience can be constructed as part of a life story. Jenni felt that she exceeded herself and the memory gave her strength. Her childbirth experience was empowering for her, and Jenni's answer implies that it affects her self‐confidence positively. Some participants also wrote about their experience after childbirth.

#### 3.3.2 Childbirth as a relational event

Childbirth as a relational event was described through two lower‐level themes: (1) relationships with family and known people and (2) relationships with professionals. The first theme consisted of subthemes of relationship with the baby, relationship with partner, relationship with another familiar person and becoming a family. Katriina (33 years old, first child) wrote:

I was relieved that I gave birth before the hospitals placed stricter restrictions considering the presence of supporting persons during labor because of the coronavirus. I received a lot of support from my husband, and I experienced that our relationship was strengthened. Maybe a birth that went well also helped me to fall in love with the baby straight away. The first moments as a family were very meaningful.

Katriina suggested that childbirth is a family event, one which starts between partners and ends in meeting the baby. The partner has a ‘supporting’ role during labour, but when a baby is born, a family is also born. This answer centred around family members, but caregiving personnel were also mentioned in many answers. The second theme encompassed relationships with all care personnel during pregnancy, childbirth and postpartum. For example, in the following experience of Hanna‐Leena (38 years old, first child), negative aspects of childbirth were described through relational themes:

The beginning and the opening phase of birth went very well, even though they took hours. […] In the beginning of the pushing stage, though we were left totally alone with my husband, because the ward was full of birthing people and the midwives were with them. The heartbeat of the child became weaker a couple of times, and it took a long time before we got a midwife to come. We started to be afraid for the well‐being of the child. The child did not descend [in the pelvis], and they did not intervene because of the hurry of midwives before 2 h. Finally, we ended up in fierce ventose extraction. I thus feel that we were well cared for until pushing stage, and I wasn't afraid, but then we were left alone in the room for a long time, and we both with my husband had to be afraid of weakening of the heartbeat and if somebody comes quickly to help if they weaken again. I was badly torn, which was one of my worst fears beforehand. The end of the birth was thus some kind of a disappointment, and I would have hoped for better support for it.

In Hanna‐Leena's answer, feelings of loneliness and mistrust were apparent. She described that the parents were afraid for the condition of the baby and in need of support, care, and reassurance. However, Hanna‐Leena and her partner were left alone because all the midwives were caring for others. A subtle hint of competition over midwives' care could also be inferred from her answer, which suggests that relationship with other mothers may also be a meaningful part of the childbirth experience.

#### 3.3.3 Childbirth as a medical event

Childbirth as a medical event consisted of four lower‐level themes: (1) obstetric story; (2) condition of the baby; (3) condition of the mother and (4) the care system. An obstetric story consists of several subthemes: the duration of the childbirth stages and childbirth events, parity, gestational age, complications, interventions, pain relief, size of the baby, mode of delivery and ‘natural birth’. Lilja (34 years old, first child) described:

My labor lasted about 11 h, beginning from arrival at hospital at 1000 h noon for a scheduled induction and ending at the birth of my daughter at 2100 h in the evening. I did not have contractions before they ruptured the membranes. The labor was fairly quick even though contractions continued quite a while, so I got an epidural [anaesthesia] for pain relief. I suffered from slight dryness because nothing kept inside, and I was not allowed to drink for the risk of the bladder impeding the labor. The child was 3.9 kg, [and it] came head first with ventose [extraction]. […] I would imagine that the labor went in a usual way.

In Lilja's childbirth story, the labour was described through institutional rhythms and hospital conventions. The details were obstetric, such as interventions, length of labour, mother's dryness and the size of the baby. Lilja described that the labour went ‘in a usual way’, indicating that the ‘usual’ is the same as the obstetric interpretation of the events.

The baby's condition was most often mentioned when there were worries, for example, when the heartbeat of the baby weakened. Moreover, many mothers mentioned how their babies were doing at birth, with a few also mentioning their babies' Apgar scores. The mother's condition was mentioned most often in relation to events after birth as a general appraisal of the physical and sometimes emotional condition of the mother.

The fourth theme, ‘the care system’, consisted of the following subthemes: transitions, positive and negative aspects of care and controlling power of the care system. Transitions were described, as leaving for the hospital, being admitted to the labour ward, moving to the postnatal ward, and leaving for home with the baby. For example, Sanna (36 years old, third child) described: ‘At the arrival at the hospital, I was 7‐cm open, and we were allowed straight to the labor room’. The choice of the words ‘being allowed’ indicated that Sanna positioned herself in a submissive role to the hospital, which had its own conventions and rules to determine labour progress and provide care.

Oftentimes, mothers described positive and negative aspects of care in their stories. They were distinct from relational aspects and consisted of evaluations of the quality of care and restrictive aspects of the care protocol. Furthermore, some mothers had descriptions of obstetric violence in their stories. Säde (23 years old, first child) wrote:

The birth was a hard experience, especially the pushing stage, because I was not listened to but instructed, and I felt powerless and inferior. The experience was also hard because the contractions had continued for 4 days before our firstborn was born. Also, the midwives did not tell that they were going to do an episiotomy; I realized myself when they spoke together about anaesthesia.

Säde described that she experienced being instructed in a harsh way during the pushing stage, which made her feel powerless and inferior. Säde's description can be interpreted as indicative of the power imbalance between hospital staff and labouring women. In her case, this translated into harsh treatment. Furthermore, doing an episiotomy without consent represented obstetric violence. In our data, a few childbirth stories indicated incidents of obstetric violence. Even though the stories were short, some women wrote about mistreatment and control. Some descriptions of controlling power of the hospital were concerning restrictions in place because of the coronavirus, whereas others were unrelated to the pandemic.

### 3.4 Childbirth experiences and changes in self‐esteem

The third research question was how women's descriptions of their childbirth experiences differed between different groups according to changes in self‐esteem during the first year after birth. To answer this question, we investigated further the open‐ended answers of the mothers who had a statistically significant change in self‐esteem between T2 and T3. The individual change was compared with randomly varied change due to measurement error. Change should be at least two times the standard deviation of random measurement error. We aimed to investigate whether their childbirth stories could explain why their self‐esteem changed for better or worse during the following year. Six participants demonstrated an increase in self‐esteem, and eight demonstrated a decrease in self‐esteem at that time (in total, *n* = 14). The mothers' qualitative descriptions of their childbirth experiences were markedly varied. Changes in self‐esteem could not be directly related to positive and negative childbirth stories, but both types of experiences were reported in both groups. However, the mothers' descriptions did vary in their contents and the sense of accomplishment or failure that they encompassed.

### 3.5 Positive experience and increasing self‐esteem: ‘Overall positive experience’

Four participants had a positive childbirth experience and a statistically significant increase in self‐esteem between T2 and T3. They all described obstetrically uncomplicated births, even though two of them experienced labour induction or augmentation. One participant described her childbirth as ‘natural’, and three of the four participants mentioned that their childbirths were quick. All participants in this group described that their childbirths met their expectations. Anni (35 years old, first child) wrote:

Overall, the birth was a positive experience for me. I was hoping that in labor, everything would advance as naturally as possible and at its own pace, if possible. This was mostly realized; only contractions needed to be restarted medically when they declined after having started.

In this group, all mothers described their childbirths mainly as lived experiences. One mother mentioned that events advanced mainly naturally and at their own pace, even though contractions were augmented medically. In these descriptions, the obstetric details were described as they were experienced, emphasizing the connection between events and experiences. One characteristic to these stories was also the participants' ability to flexibly define their births in favourable terms, even though some aspects were not realized according to the women's wishes (i.e. induction or augmentation of labour). The participants defined the unplanned aspects of their experience as minor distractions in an overall positive experience.

### 3.6 Negative experience and increasing self‐esteem: ‘Harder and more painful than I expected’

Three participants demonstrated a negative childbirth experience and a statistically significant improvement in self‐esteem between T2 and T3. All participants in this group described their childbirths as more difficult than expected or, otherwise, not meeting their expectations. However, they also described positive aspects of their childbirths. Maarit (29 years old, first child) wrote:

The labor was long and harder and more painful than I expected. I got all the pain relief, but, nevertheless, the pain was bad. I did not really sleep for a couple of days or eat for 1 day. Fortunately, the midwives were lovely, and the labor was not only a negative experience thanks to them. I was disappointed, however, because the experience was mainly hard and painful and not the kind of empowering that I had wished. Remembering the labor only makes me weep. I stayed at hospital for 36 h, and the labor lasted for 13 h, pushing 50 min.

Maarit described her birth as more difficult and painful than expected. Many of her expectations were violated mainly because the labour was so long and painful. She described her experience mainly as a lived experience, with some obstetric details and praise for the midwives as lovely supporting persons, representing the main category of childbirth as a relational event.

The birth stories in this group were told either as a combination of the three main themes but centralizing on childbirth as a lived experience or as a medical event. None of the participants attributed the difficulties explicitly to anyone or anything, except for one participant who expressed that she was bad at enduring pain. However, the main difficulties in her description were elsewhere, and she described a complication that upset her. Overall, these descriptions paint a picture of somehow complicated or hard childbirths; however, all three mothers also described something positive in their experiences. The readers got the impression that these births were difficult but not extremely traumatic to women due to positive factors.

### 3.7 Negative experience and decreasing self‐esteem: ‘Terrible’

Two of our participants demonstrated a negative experience of childbirth and a statistically significant reduction in self‐esteem between T2 and T3. Both described an extremely difficult childbirth experience, and both gave birth through a caesarean section. Paula (34 years old, first child) wrote:

Terrible. My only wish was that I would not need to go to C‐section, I was prepared for pain, and I think that my expectations for birth were realistic. However, the labor did not advance after the beginning, the heartbeat of the child dropped once greatly, and I developed a fever (infection of the membranes). Pain relief was insufficient (the epidural block worked only on one side). After about 24 h, the child was born by C‐section, where pain relief was inadequate (the spinal block was inserted in the epidural space). After the exertion of the child, they attempted to strongly medicate the pain for 30 minutes while the operation continued, but the operating pain was severe, and, finally, I was anaesthetised unconscious. The only positive thing was that I did not experience the C‐section as preposterous, but there were indications to proceed into it, and there was no sense of hurry. The event itself and prolonging and fear for the child's well‐being and operating without adequate anaesthesia were not nice.

Paula wrote that her childbirth expectations were realistic and that her ‘only wish’ was to avoid a caesarean section. However, she defined her childbirth experience as ‘horrible’ and described a violation of numerous implicit expectations: the labour did not advance, worry concerning the baby's condition and development of an infection. Anaesthetic pain relief did not sufficiently block her pain, and the childbirth ended in a caesarean section after a long time in labour. Finally, she had to undergo operation under general anaesthesia because of the continuing pain during the caesarean section. Paula did not attribute the difficulties clearly, and the reader is left wondering whether she blames someone or something for the labour that did not advance as expected.

Both birth stories in this group described births that can be understood as potentially traumatic events. Both participants expressed concern for the well‐being of the baby, and both experienced a threat to their own integrity. In the example above, Paula experienced severe pain over a prolonged time. In the other story in this group, Vilja (28 years old, first child) described having a deep sense of failure: ‘I felt that I had let down all people who were present because I was not able to push the baby out. Even now feelings of guilt pass my mind sometimes’. It is possible that these childbirth experiences might have altered the participants' self‐perceptions or perceptions of the world through traumatization. These birth stories were told through all three main themes, suggesting that the participants had an overall negative experience at all possible interpretations or frameworks.

### 3.8 Positive experience and decreasing self‐esteem: ‘All went fairly quickly and well’

Six participants experienced positive childbirths and had a statistically significant decrease in self‐esteem between T2 and T3. Though rated as mainly positive, all but one participant in this group described experiences that included both positive and negative elements. Three of six participants in this group described interventions: induction of labour, caesarean section and instrumental birth (vacuum extraction). Marika (27 years old, second child) wrote:

Daughter was born at gestational age 40 + 6. The labor started naturally and lasted for about 8 h. At the beginning, I went without medical pain relief. At 7 cm, the pain started to be so severe that we tried a spinal block. The first time did not work, so 1 h after, at 9 cm, it was inserted again, and then it worked. Until that moment, the contractions came every couple of minutes, and they were really fierce (my whole body trembled because of the force of the contractions). The end went quickly. Pushing took 15 min and felt controlled. I liked this birth because it did not last too long, and I did thus not get too tired, but I could well follow the course of labor and concentrate on the experience.

Marika shared her experience as both a medical event and lived experience. The main positive factors were related to her experiences, such as feelings of control, conserving her energy, and being able to concentrate. However, this was described as enabled by an obstetrically uncomplicated birth that advanced well. Marika's story is about something happening to her, not about her doing something.

In this subgroup, mothers most often told their birth stories through a combination of lived experience and obstetric event or a combination of all three themes. Even though their experiences were positive, most mothers in this group did not merit themselves. Instead, they described that giving birth ‘did not last too long’, ‘advanced fairly quickly’, ‘all went fairly quickly and well’, ‘avoided complications’ and ‘this time, I was met and heard’. Positive aspects were interpreted as obstetric and sometimes relational, not intrinsic to the mothers themselves. The only exception in this group for this type of birth story is the description of Ellen (27 years old, first child). She described active agency and pride of her own capacities: ‘I am proud of my own actions, because they kept the labor advancing’ and ‘I knew how to push and gave my best. I am very happy that I concentrated fully on every moment and gave my best without panicking’. Ellen had a very high self‐esteem at all time points, even though it decreased between T2 and T3. In her case, it was difficult to interpret that her childbirth experience was associated with decreased self‐esteem. Rather, it was probably due to other factors.

## 4 DISCUSSION

The aim of the present research was, first, to determine whether childbirth experience predicts changes in self‐esteem during the first year after childbirth. As a result of the statistical analyses, we found that childbirth experience as measured by the Delivery Satisfaction Scale statistically predicted changes in self‐esteem during the first year after childbirth. The more positive the childbirth experience, the greater the increase in self‐esteem; conversely, the more negative the childbirth experience, the greater the decrease in self‐esteem. The results were not dependent on parity or other background variables. This finding is in line with previous research (e.g. Laney et al., 2014; Reisz et al., 2015). For example, Parratt (2002) concluded in a literature review on the effects of childbirth on women's sense of self that childbirth can have either positive or negative effects. Though not specifically concentrating on self‐esteem, Parratt's ‘sense of self’ encompasses many aspects contributing to healthy self‐esteem, such as feelings of control and empowerment, an ability to listen to oneself empathically, effective communication and forming trusting relationships. Studies that concentrated on positive experiences (e.g. Olza et al., 2018) have suggested that childbirth can be empowering for women. On the other hand, the traumatic experiences of childbirth have been identified to alter women's self‐perceptions negatively (e.g. Byrne et al., 2017).

The second aim of this research was to investigate how mothers describe their childbirth experiences when answering an open‐ended question. The findings of our qualitative analysis demonstrated that women understand their childbirth experiences through three main themes: childbirth as a lived experience, childbirth as a relational event and childbirth as a medical event. Even though responses were generally quite short, many of them included several subthemes and often two or three main themes. This finding confirms most of the extant research suggesting that childbirth experiences are multifaceted (see, e.g. Dencker et al., 2020). However, it also contributes to the current understanding by showing that most women consider their childbirth experiences from multiple perspectives or ideological standpoints. The contents of the overarching themes resemble partly those of Davis‐Floyd (2001), most notably in case of childbirth as a medical event, which resembles Davis‐Floyd's technocratic model. Many women in our data wrote their birth stories as medical events, including surprisingly many obstetric details. Women appear to have internalized the normative biomedical model of birth (Preis, Eisner, et al., 2019) to such extent that they tell their stories through that lens. However, our findings suggest that for Finnish women, their lived experiences and the relational aspects of childbirth were the other main aspects of birth, which both represent the humanistic paradigm of birth in Davis‐Floyd's (2001) analysis. Representing other frameworks apart from the medical model, these overarching themes challenge the normative technocratic model of birth. Holistic interpretations appear to be absent in our findings, possibly because of our research methodology (short survey answers) or the cultural differences between Finland and the United States.

The third aim of the present research was to determine how women's descriptions of their childbirth experiences differed between different groups on the basis of changes in self‐esteem during the first year after birth. Our findings suggest that the effect of childbirth experience on self‐esteem might be straightforward only for extreme cases, that is, women who had an exceptionally positive childbirth experience and women who experienced their childbirths as traumatic. One explanation for the negative impact is that traumatization affects self‐concept (Byrne et al., 2017). However, the effects of both positive and negative experiences may be explained by cognitive factors. Schneider (2010) suggested that women evaluate themselves on the basis of their labor performances, much like birth were a test of their values. In her interpretation, one's sense of self can be altered when the actual childbirth experience does not match expectations. Our findings are in line with the effect of feelings of failure (Schneider, 2018) but also suggest that positive experiences may contribute to better self‐esteem.

Women with mixed experiences were among those who had a trajectory of increasing self‐esteem and those whose self‐esteem decreased. In case of mixed experiences of childbirth, it is probably factors other than childbirth experience per se that could explain changes in self‐esteem during the following year. This is encouraging because it means that childbirth experiences do not always determine self‐esteem development. This is also somewhat surprising compared with the findings of Schneider (2010): in her data, 60% of women experienced feelings of failure in relation to childbirth, whereas in our data, 12% of women experienced a change in self‐esteem in the postpartum year. This discrepancy may imply that in most cases, feelings of failure do not affect self‐esteem in the long run. One possible interpretation is that it depends on the women as to what aspects of the childbirth experience are most important for their self‐esteem. Contrasting positive and negative aspects of childbirth may also sometimes cancel out each other, that is, partially diminishing and partially supporting one's self‐esteem.

The results of our study are somewhat confusing for the participants, whose childbirth experiences were positive and who, nevertheless, showed a decrease in self‐esteem in the postpartum year. All but one participant in this group had a trajectory of (statistically non‐significantly) increasing self‐esteem from T1 to T2 and decreasing self‐esteem from T2 to T3. Although different trajectories in self‐esteem development could not be identified in the statistical analyses owing to the small sample size, one explanation for these unexpected findings could be that a positive childbirth experience might already have affected self‐esteem at T2: that is, compared with that at baseline, self‐esteem had increased but decreased back to baseline during the following year. This might also be explained by the way that the participants described their childbirths. The positive aspects they described most often were obstetric factors such as short delivery duration, and they did not merit themselves for it. It is probable that obstetric factors were perceived as uncontrollable and, in this case, represent good luck rather than one's own capacities.

Another interesting group with unexpected findings was those of women who had a negative experience of birth and whose self‐esteem increased in the following year. Those women had DSS scores situated in the middle of the scale, that is, clearly more negative than the scores of most women in our data. The main difficulties described in the birth stories were obstetric and not interpreted through one's own capacities (or lack thereof). Instead, the participants described either unexpected complications or unexpectedly long delivery duration perceived as difficult and painful. These kinds of stories can be interpreted as hero stories: these women were faced with enormous challenges that they successfully went through. This kind of interpretation is likely to support one's self‐esteem even in cases where some weaknesses are attributed to oneself.

Overall, our study contributes to existing knowledge by suggesting that self‐esteem development in the perinatal period is a complex issue, with considerable individual variation. Childbirth experience seems to contribute to the development of self‐esteem for some women but appears to be unrelated or not so significant for others. However, the findings of the present research are unique because of the longitudinal study design and focus on basic, not domain‐specific, self‐esteem in the perinatal period. Furthermore, a mixed methods approach is useful for complex issues such as psychological development in the transition to motherhood. In our research, this method allowed the examination of individual variation in self‐esteem development and childbirth experiences. Despite its strengths, the present study also has limitations. First, the sample size was small, which made it unreasonable to statistically divide the sample into subgroups. For that reason, opportunities for triangulation were limited. Second, the sample was homogenous, which limits the generalizability of the results. For example, less advantaged women might generally have lower self‐esteem than the highly educated women in our sample, and this difference might have produced even more inter‐personal variability in the results. Third, despite the longitudinal study design, self‐esteem was measured only three times during data collection, and possible daily fluctuations were not accounted for. Thus, we could not evaluate the role that the stability or reactivity of self‐esteem plays in our results. Finally, due to the global pandemic, our results may be specific to this stressful situation and need to be confirmed in other studies. In future research, larger and more heterogenous samples are needed, and daily fluctuations in self‐esteem should be accounted for.

## 5 CONCLUSION

In line with previous research (Byrne et al., 2017; Laney et al., 2014; Olza et al., 2018), our findings support the hypothesis that childbirth experience may affect self‐esteem development for better or worse in the year after birth. For women who find their childbirths especially satisfying, the experience may serve as an important personal resource. Therefore, care systems should be adapted to support positive experiences for all women, for example, by developing continuity‐of‐care models within the care systems (Donate‐Manzanares et al., 2021) and respectful, women‐centred care. Women's own interpretations of their childbirth experiences should be encouraged, which may facilitate the recognition of birth as a multifaceted experience and build self‐compassion. More attention should be directed at women who experienced traumatic childbirths because they are at risk of negative changes in self‐esteem. Women should be screened for traumatic childbirth experiences in postpartum care and offered adequate support. More research is needed to confirm the results in bigger and more heterogenous samples and to better understand variations in self‐esteem development in postpartum women.

## AUTHORS' CONTRIBUTIONS

All authors have agreed on the final version and meet at least one of the following criteria (recommended by the ICMJE*):

1. substantial contributions to conception and design, acquisition of data, or analysis and interpretation of data;
2. drafting the article or revising it critically for important intellectual content.

## FUNDING INFORMATION

This research received no specific grant from any funding agency in the public, commercial, or not‐for‐profit sectors.

## CONFLICT OF INTEREST

No conflict of interest has been declared by the author(s).

### PEER REVIEW

The peer review history for this article is available at https://publons.com/publon/10.1111/jan.15468.

## Supporting information


Supplementary 1

## Data Availability

The data that support the findings of this study are openly available in JYX data archive at https://doi.org/10.17011/jyx/dataset/81710.
