# Supplementary material for: Passing the test of motherhood? Self‐esteem development and birth experience in the transition to motherhood: A longitudinal mixed methods study in Finland
Source: J Adv Nurs. 2022 Oct 17;78(12):4246–60. doi: 10.1111/jan.15468 (PMC9828506; doi:10.1111/jan.15468)
Supplement: Supplementary file 1 — Supplementary 1 [file JAN-78-4246-s001.docx]

**Supplement 1.** Measures.

Delivery Satisfaction Scale (Finnish/English)

1 = very much, 5 = not at all

1. Oliko synnytys sinulle myönteinen kokemus?

Was childbirth a positive experience for you?

1. Saitko henkilökunnalta riittävästi tukea?

Did you receive enough support from the hospital staff?

1. Saitko puolisoltasi/tukihenkilöltä riittävästi tukea?

Did you receive enough support from your partner/supporting person?

1. Oliko synnytys kivulias?

Was your childbirth painful?

1. Oliko saamasi kivunlievitys riittävä?

Was the pain relief sufficient?

1. Tunsitko olosi turvalliseksi synnytyksen aikana?

Did you feel safe during labor and birth?

1. Saitko vaikuttaa haluamallasi tavalla synnytyksen hoitoon?

Could you affect the management of labor as you wished?

1. Ottiko henkilökunta liikaa ohjat omiin käsiinsä synnytystä hoitaessaan?

Did the hospital staff take too much control over the management of labor?

Items 1, 2, 3, 5, 6 and 7 are reverse scored.

Rosenberg Self-esteem Scale

1 = strongly disagree; 5 = strongly agree

1. Tunnen, että minulla on arvoa ihmisenä vähintään yhtä paljon kuin muillakin.

I feel that I am a person of worth, at least on an equal plane with others.

1. Aina silloin tällöin ajattelen, ettei minussa ole mitään hyvää.

At times I think I am no good at all.

1. Uskon, että minulla on monia hyviä ominaisuuksia.

I feel that I have a number of good qualities.

1. Pystyn tekemään asioita yhtä hyvin kuin muutkin ihmiset.

I am able to do things as well as most other people.

1. Tietysti tunnen itseni hyödyttömäksi ajoittain.

I certainly feel useless at times.

1. Minulla on myönteinen käsitys itsestäni.

I take a positive attitude toward myself.

1. Kaiken kaikkiaan minun on tunnustettava, että olen epäonnistunut monessa asiassa.

All in all, I am inclined to feel that I am a failure.

1. Olen kaiken kaikkiaan tyytyväinen itseeni.

On the whole, I am satisfied with myself.

1. Minulla ei ole paljoakaan, josta voisin olla ylpeä.

I feel I do not have much to be proud of.

1. Toivoisin voivani kunnioittaa itseäni enemmän.

I wish I could have more respect for myself.

Items 2, 5, 7, 9 and 10 are reverse scored.
